# Residual Splenic Volume after Main Splenic Artery Embolization is Independent of the Underlying Disease

**DOI:** 10.5334/jbsr.2068

**Published:** 2021-04-06

**Authors:** Johannes Devos, Lawrence Bonne, Sandra Cornelissen, Walter Coudyzer, Wim Laleman, Chris Verslype, Willem-Jan Metsemakers, Geert Maleux

**Affiliations:** 1 Department of Radiology, University Hospitals Leuven, Leuven, Belgium; 2 Department of Gastroenterology and Hepatology, University Hospitals Leuven, Leuven, Belgium; 3 Department of Traumatology, University Hospitals Leuven, Leuven, Belgium

**Keywords:** splenic artery, embolization, splenic volume, computed tomography

## Abstract

**Purpose:**

To assess the safety and efficacy of main splenic artery embolization. To assess the potential difference post-embolization of the residual splenic volume in patients embolized for trauma versus those embolized for (pseudo)aneurysms.

**Materials and Methods:**

A retrospective analysis was performed on a cohort of 65 patients (36 males) who underwent pre- and post-embolization computed tomography. Patients’ demographics, pre- and post-interventional medical and radiological data were gathered. Splenic volume calculations were semi-automatically performed via a workstation. Patients with splenic aneurysms or pseudoaneurysms of the main splenic artery (group 1) were compared to those with splenic rupture (group 2) using Wilcoxon rank tests.

**Results:**

The main indications for splenic artery embolization were splenic rupture (n = 22; 34%) and splenic pseudoaneurysm (n = 19; 29%). The technical success rate was n = 63; 97%. The procedure-related complication rate was n = 7; 11%, including abscess formation (n = 5; 8%), re-bleeding (n = 1; 1.5 %) and pseudoaneurysm re-opening (n = 1; 1.5%). The overall 30-day mortality was n = 7; 11%.

Median follow-up for groups 1 and 2 was 1163 days (61–3946 days) and 702 days (43–2095 days) respectively. When processable (n = 23), the splenic volume in group 1 (n = 7) was 311 cm^3^ and 257 cm^3^ (p = 0.1591) before and after embolization respectively, and in group 2 (n = 16) it was 261 cm^3^ and 215 cm^3^ (p = 0.4688), respectively.

**Conclusions:**

Main splenic artery embolization is efficacious, with low procedure-related complication and 30-day mortality rates. No significant differences in residual post-embolization splenic volume were found between patients treated for splenic rupture versus those treated for splenic arterial (pseudo)aneurysm.

## Introduction

A variety of clinical disorders, including focal lesions of the main splenic artery and splenic parenchymal disorders can be managed by catheter-directed splenic artery embolization (SAE) as an alternative to surgical splenectomy [1]. Additionally, main splenic artery coil-embolization can preserve substantial residual splenic tissue and function [2,3], which may also result in a better long-term clinical outcome. The anatomical and physiological mechanism for preservation of splenic parenchyma and function after coil-embolization is related to the rich collateral arterial supply from various branches, including left gastric, left gastroepiploic, and pancreatic branches to the distal main splenic artery and the first branches.

We thus hypothesized that splenic volume reduction after main splenic artery embolization would not be influenced by the underlying disease. Therefore, safety and efficacy of main splenic artery embolization for various indications is analyzed in this study, along with post-embolization splenic volume changes in patients embolized for splenic trauma versus those embolized for main splenic artery (pseudo)aneurysm.

## Materials and Methods

### Study design and study population

This is a retrospective, single center, observational study including a cohort of patients who underwent main SAE in the interventional radiology department of an academic, tertiary care center, from January 2000 until December 2016. This study was approved by the local ethics committee. Patients’ pre-interventional data, including patients’ medical history and radiological pre-interventional investigations, as well as procedural data and clinical and radiological follow-up data were gathered from the institutional electronic medical records. Pre-, peri- and post-interventional radiological data were analysed from a PACS system (IMPAX, Agfa Gevaert, Mortsel, Belgium) including splenic volume calculations (SyngoVia, Siemens, Forchheim, Germany).

### Angiographic embolization procedure

SAE procedures were performed under local or general anaesthesia depending on patient’s general status. After gaining access to the right common femoral artery, a 4 French (F) vascular sheath was inserted. With use of a 4F Simmons I catheter (Performa, Merit Medical, South Jourdan, UT, USA or Glide Cath, Terumo Europe, Leuven, Belgium) or a 4F Cobra catheter (Slip Cath, Cook Medical, Bjaeverskov, Denmark) the celiac trunk was catheterized. The main splenic artery was superselectively catheterized with use of a microcatheter (Progreat 2.7, Terumo Europe, Leuven, Belgium or Cantata 2.5, Cook Medical, Bjaeverskov, Denmark or Direxion 2.8, Boston Scientific, Natick, MA, USA). Embolization was performed using pushable microcoils (Target, Boston Scientific, Natick, MA, USA or Micro-Tornado and MicroNester, Cook Medical, Bjaeverskov, Denmark) or in combination with a mixture of cyanocrylate (Histo-acryl, B. Braun, Melsungen, Germany) and ethiodized oil (Lipiodol, Guerbet, Aulnay-sous-Bois, France). In case of focal vascular lesion of the main splenic artery (true aneurysm or pseudoaneurysm), coil embolization was performed by placing micro-coils distally and proximally to the vascular lesion. In case of a splenic parenchymal rupture, micro-coils were placed in the main splenic artery, proximally to the first bifurcation in the hilum of the spleen. A completion splenic artery angiogram with manual injection through the microcatheter and automated pump injection through the diagnostic 4F catheter were performed before removal of the vascular sheath and manual compression in the groin.

Technical success of the embolization procedure was defined as successful transcatheter placement of the embolics in the main splenic artery with angiographic occlusion of the coiled segment and complete disappearance of the underlying vascular lesion on completion angiography.

### Splenic volume calculation and patients’ follow-up

Patients’ clinical and radiological follow-up data were gathered from the institutional electronic medical records and calculations were performed on patients’ final follow-up imaging. Post-interventional radiological data were analysed from a PACS system (IMPAX, Agfa Gevaert, Mortsel, Belgium), including a semi-automated splenic volume calculation tool (SyngoVia, Siemens, Forchheim, Germany). Briefly, enhancing splenic volumes were manually delineated on each axial slice (3 mm thickness) and afterwards, total enhancing splenic volume was automatically computed by the software. Additionally, a comparative analysis of the residual, viable (= contrast-enhancing) splenic volume after embolization was performed between patients with a pre-interventional normal spleen (group 1) and patients with a pre-interventional injured spleen related to splenic parenchymal rupture (group 2).

### Statistical analysis

Descriptive statistics were used to characterize the patient demographics and to evaluate the 30-day mortality rate. Comparison of the splenic volume prior to and following main SAE was performed using the Wilcoxon signed rank test.

## Results

### Demographic data

The study cohort included 65 patients with a mean age of 53 years (range: 17–89 years); 36 patients were male (55%) and 29 were female (45%). Indications for main SAE are summarized in ***Table 1*** and mainly include splenic rupture (group 1, n = 22; 34%) and pseudoaneurysm (group 2, n = 19; 29%). The clinical symptoms at the time of embolization were hemodynamic instability related to overt bleeding (n = 23; 35%), abdominal pain (n = 25, 38%) or none (n = 20; 31%). In case of an underlying main splenic artery (pseudo)aneurysm, the location within the main splenic artery, size, and associated clinical symptoms are summarized in ***Table 2***.

**Table 1:** Indications for main splenic artery embolization.


INDICATION	NUMBER	%

Splenic rupture	22	34%

Traumatic	12	18%

Coagulopathy	4	6%

Leukaemia	2	3%

Postoperative	2	3%

Pancreatitis	1	1.5%

Idiopathic	1	1.5%

Pseudoaneurysm	19	29%

Pancreatitis	15	23%

Postoperative	3	5%

Traumatic	1	1.5%

Aneurysm	12	18%

Focal extravasation from main splenic artery	9	14%

Postoperative hemorrhage	6	9.5%

Pancreatitis	3	5%

Splenomegaly	2	3%

Preoperative	1	1.5%


**Table 2:** Anatomic characteristics and associated symptoms of the (pseudo)aneurysms.


	ANEURYSM	PSEUDOANEURYSM

Total number (n = 31)	12 (38.7%)	19 (61.3%)

Symptomatic patient	2 (17%)	6 (32%)

Location		

Proximal MSA	2 (17%)	2 (11%)

Middle MSA	5 (42%)	9 (47%)

Distal MSA	5 (42%)	8 (42%)

Mean diameter (mm)	30.5 mm (16 mm–45 mm)	41.1 mm (22.7 mm–59.5 mm)


MSA: main splenic artery.

### Embolization procedure

**Figure 1:**
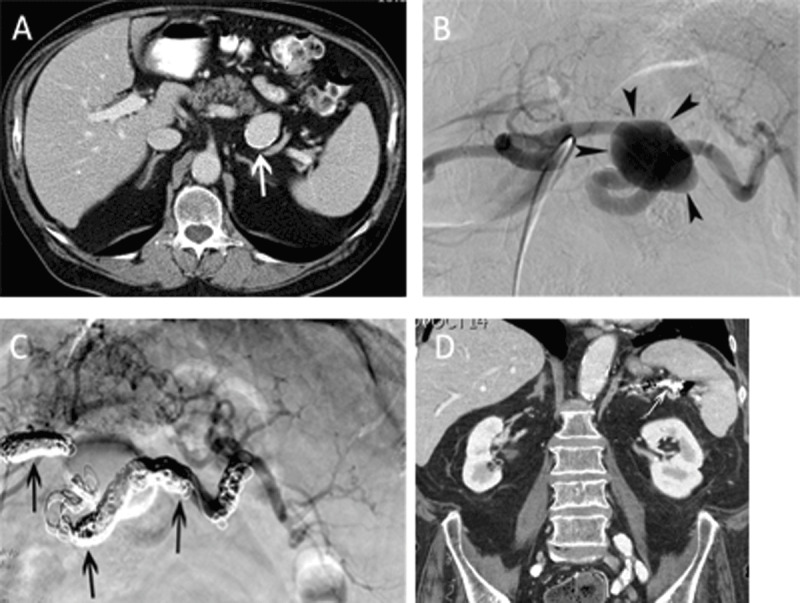
Main splenic artery embolization for atherosclerotic aneurysm. **(A)** Contrast-enhanced CT-scan in a 74-year-old man revealed an asymptomatic, atherosclerotic aneurysm (white arrow) with a maximal diameter of 37 mm. The total splenic volume was 418 ml. **(B)** Selective angiography of the celiac trunk confirmed the saccular aneurysm (arrowheads) in the middle third of the main splenic artery. **(C)** Completion angiography after coil embolization (arrows) demonstrated exclusion of the aneurysm and reinjection of the intrasplenic arteries through gastric collaterals. **(D)** Follow-up CT-scan 5 years after coil embolization revealed an homogeneously enhancing spleen with a total volume of 264 ml.

**Figure 2:**
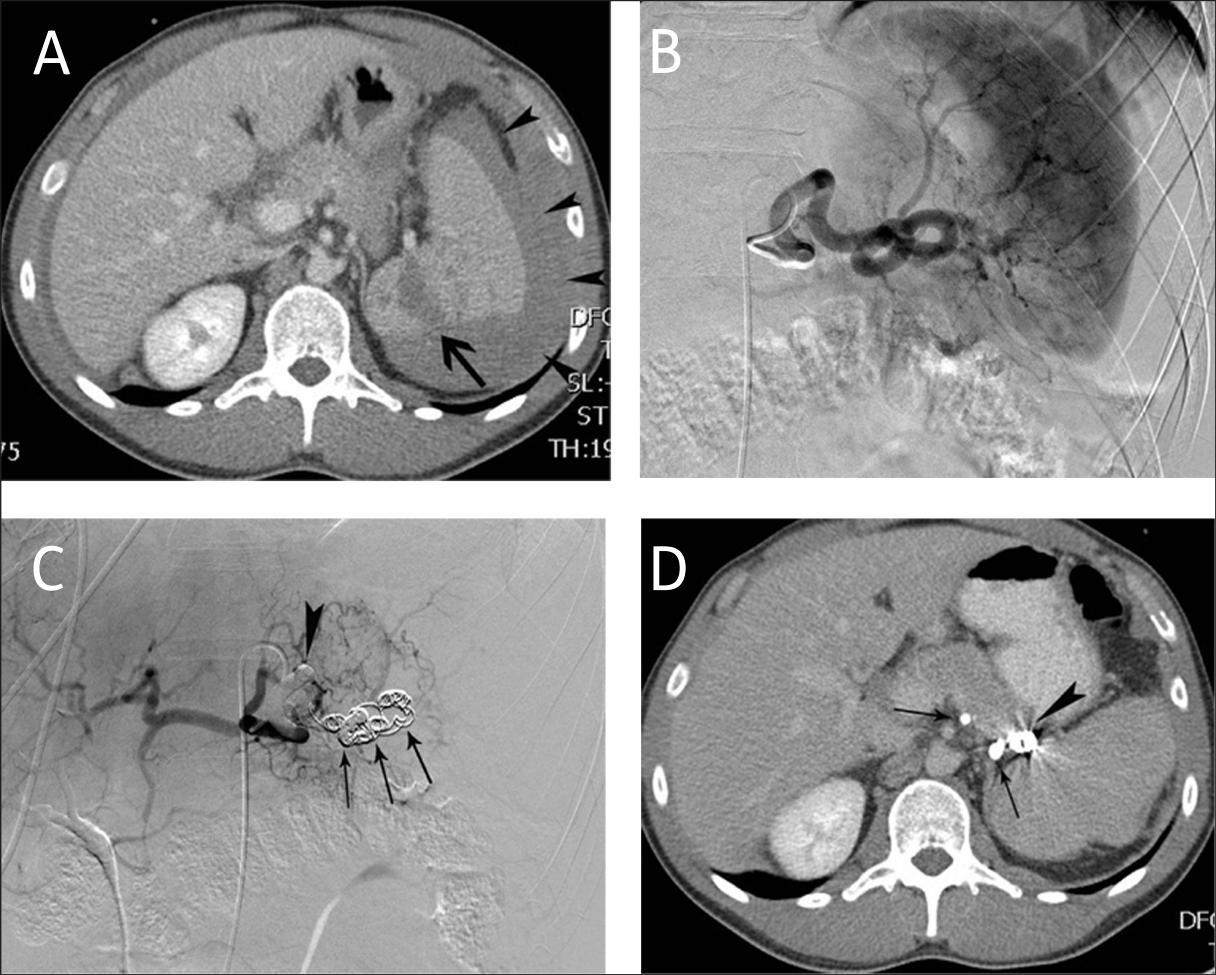
Main splenic artery embolization for trauma. **(A)** CT-scan in a 27-year-old man after a traffic accident revealed splenic laceration (arrow) and a perisplenic hematoma (arrowheads). The volume of the spleen was 270 ml. **(B)** Corresponding selective splenic angiography did not reveal contrast extravasation. **(C)** Main splenic artery embolization was performed with coils (arrows) and glue (arrowhead). **(D)** Follow-up CT-scan 8 months later demonstrated the glue (arrows) and coils (arrowhead) in the splenic artery. The total splenic volume was 290 ml.

The embolization procedure was performed under local (n = 53; 81.5%) or general anaesthesia (n = 12; 18.5%). In 49 out of 65 procedures (75%) micro-coils were the only embolics used (***Figure 1***); in seven (11%), a mixture of cyanoacrylate glue and ethiodized oil was used and in the remaining nine (14%), a combination of micro-coils and glue was used (***Figure 2*** and ***Table 3***).

**Table 3:** Distribution of embolics among indication for embolization.


	ETIOLOGY OF SPLENIC ARTERY DISEASE					

	ANEURYSM	PSEUDO- ANEURYSM	SPLENIC RUPTURE	HEMORRHAGE	SPLENOMEGALY	OTHER

Embolic material						

Glue	1	3	2	1	0	0

Glue + microcoils	0	2	1	2	0	0

Microcoils	9	13	19	6	1	1

Microcoils + microparticles	2	1	0	0	1	0


The embolization procedure was technically successful in 63 out of 65 patients (97%); in two patients the underlying vascular lesion was still partially visible on completion angiography. These patients presented with severe coagulopathy including deep thrombocytopenia (29 × 10^9^/L) and high prothrombin time (20.6 sec), respectively. In the latter case, the main splenic artery was completely thrombosed on follow-up computed tomography 12 days after the procedure.

In two patients, a micro-coil migrated distally into one of the segmental splenic arteries without adverse event; in another patient, there was a proximal micro-coil migration to the main hepatic artery which was managed by endovascular snaring (Goose neck microsnare Kit, EV3, Plymouth, MN, USA).

### Imaging follow-up

Follow-up imaging after main SAE was performed using various radiological techniques, as summarized in ***Table 4***. The last follow-up imaging retrieved from the institutional PACS system ranged from 1 day to 3946 days with a mean of 758 days. In 20 patients (30.1%) no radiological follow-up was found. Follow-up imaging data were available in 16 patients without pre-interventional splenic rupture and in 15 patients with pre-interventional splenic rupture. In 12 out of the patients who had immediate post-embolization processable imaging, there was >50% splenic tissue infarction.

**Table 4:** Follow-up radiological modality after main splenic artery embolization.


	BEFORE EMBOLIZATION		AFTER EMBOLIZATION	

	N	%	N	%

Computed tomography (CT)	59	90.7%	26	40%

US	4	6.1%	3	4%

Magnetic resonance imaging (MRI)	2	3.1%	2	3%


Excluding patients with imaging follow-up of less than 30 days, data were available for 23 patients, including 16 with a normal, pre-interventional spleen (group 1) and 7 with splenic rupture (group 2) (***Table 5***). There was no difference in enhancing splenic volume between patients with a splenic artery (pseudo)aneurysm (group 1) and those with a traumatic splenic rupture (group 2) (p = 0.6244). Analysing the difference in splenic enhancing volume pre- versus post-embolization showed no difference, both in group 1(p = 0.1591) and in group 2 (p = 0.4688).

**Table 5:** Imaging follow-up of enhancing splenic volume in patients with a pre-interventional normal spleen (group 1) versus patients with a traumatic splenic rupture (group 2).


	TRAUMATIC SPLENIC RUPTURE (n = 16)	SPLENIC ARTERY (PSEUDO)ANEURYSM (n = 7)

Pre-interventional splenic volume	261 ml	311 ml

Post-interventional splenic volume	215 ml	257 ml

Median follow-up (days)	702 (43–2095)	1163 (61–3946)


### Clinical outcome data

**Figure 3:**
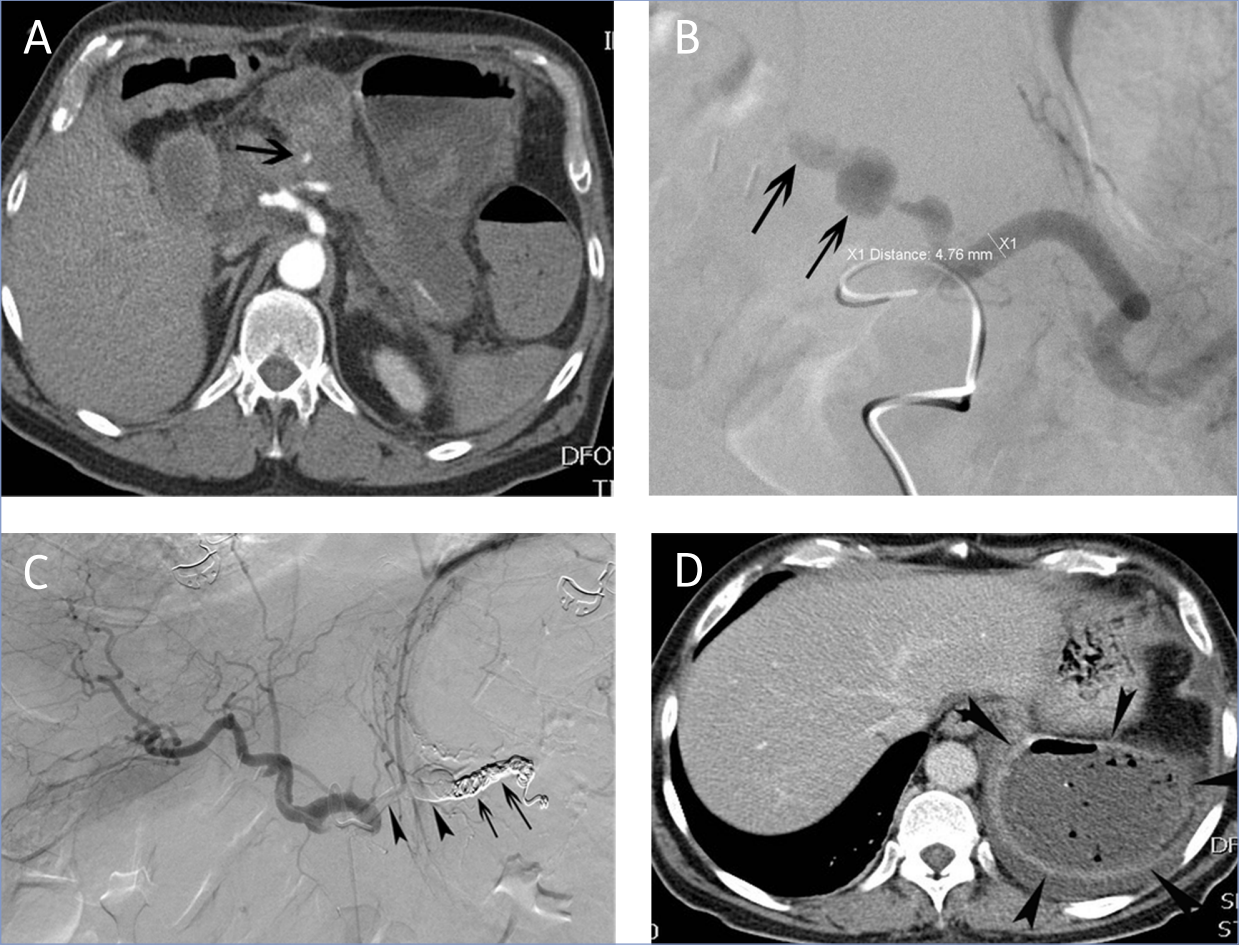
Main splenic artery embolization for postoperative hemorrhage. **(A)** CT-angiography in a 64-year-old man 19 days after pancreatico-duodenectomy revealed contrast extravasation (arrow) in the proximal part of the main splenic artery. **(B)** Corresponding selective splenic arteriography confirmed the contrast extravasation and a pseudoaneurysm (arrows). **(C)** Embolization of the hemorrhage was performed with a combination of coils (arrows) and glue (arrowheads). **(D)** CT eight days after embolization revealed multiple air bubbles in a completely necrotic splenic tissue compatible with abscess.

A minority of patients (n = 19; 29%) received prophylactic pneumococcal and meningococcal vaccination prior to the embolization. Clinical post-embolization complications were identified in seven patients (11%) and included splenic abscess (n = 5; 8%), rebleeding (n = 1; 1.5%), and pseudoaneurysm reopening (n = 1; 1.5%). Treatment of these post-interventional complications included administration of intravenous antibiotics with (n = 2) or without (n = 2) percutaneous drainage (***Figure 3***) and splenectomy (n = 1). The 30-day mortality rate after main splenic artery embolization was n = 7 (11%), related to complications of underlying acute pancreatitis (n = 4), sickle cell crisis (n = 1), multiple organ failure (n = 1), and poor general condition (n = 1).

## Discussion

This study confirms the very high technical success rate of main SAE for various indications as previously found for splenic artery aneurysms [2,4,5] and traumatic splenic rupture [6,7]. Complications related to the embolization procedure are relatively rare and mainly related to splenic necrosis and abscess [8]. In this report a total of five patients (8%) had post-embolization splenic abscess. This is in line with a study by Gaba et al. [9] who found such complication in two out of 50 patients (4%), specifically an encapsulated bacterial infection in one patient and a splenic abscess in another patient, suggesting the need for antibiotic prophylaxis before and after the embolization. Reopening of an aneurysm is uncommon after main SAE. In this cohort a repeat embolization was performed in one out of 65 patients (1.5%), which is lower than other series mentioning a repeat intervention rate of 9% [4] and 10% [9] of cases.

Finally, in two patients migration of micro-coils within the segmental arteries of the spleen was observed, which may be avoided by using a Penumbra occlusion device or vascular plugs [10].

The 30-day mortality after successful splenic artery embolization is not negligible but mainly related to the underlying disease, and not to the embolization procedure. Gaba et al. [9] found a 30-day mortality of 8% and in a multivariable analysis, renal insufficiency, pre-procedure hemodynamic instability, and pre-procedure leukocytosis seem to be prognostic factors for 30-day mortality.

Comparison of enhancing splenic volume before and after main splenic artery embolization revealed a modest volume reduction after embolization without statistical significance which is in line with the findings of Preece et al. [3]. However, these authors found, in a multivariate analysis, that distal coil pack location in the main splenic artery was the only factor significantly affecting splenic volume loss. Li et al. [2] found a significant difference in favour of patients treated with coil embolization of the aneurysmal sac with patency of the splenic artery compared to patients treated with coil embolization of the main splenic artery with complete occlusion of the artery and the aneurysm. Overall, irrespective of the indication for main SAE, residual splenic function after embolization is most often seen, which is one of the major advantages over splenectomy. Additionally, Preece et al. [3] did not find Howell-Jolly bodies persisting after embolization, which is another argument in favour of maintained splenic function after embolization.

Finally, this study has some limitations. First, this is a retrospective analysis and embolization procedures were not performed using the same technique; however, in most cases, only coils were used. Second, not all patients had the same radiological follow-up protocol and some patients were lost to follow-up. Third, although 65 patients were included in the study, the sample size of both study groups is still small. Last, only radiological factors were analysed to evaluate residual splenic function without including biochemical parameters.

In conclusion, this retrospective analysis demonstrates that main splenic artery embolization for various clinical indications is safe and efficacious, with a 10% 30-day mortality rate. Post-interventional splenic volume reveals a modest but insignificant volume loss in both patients with splenic trauma and those with splenic artery (pseudo)aneurysm, which may add weight to the medical literature in favour of catheter-directed main splenic artery embolotherapy rather than splenectomy for various clinical indications.
